# Double trouble: *Mycoplasma pneumoniae* and its unexpected complications

**DOI:** 10.1093/ehjcr/ytaf467

**Published:** 2025-09-18

**Authors:** Ai Phi Thuy Ho, Eirik Brekka Tjønnfjord, Saad Aballi, Sara Foss Debes, Ole-Christian Rutherford

**Affiliations:** Department of Cardiology, Ostfold County Hospital Kalnes, Sarpsborg 1714, Norway; Department of Emergency Medicine, Ostfold County Hospital Kalnes, Sarpsborg 1714, Norway; Department of Infectious Diseases, Ostfold County Hospital Kalnes, Sarpsborg 1714, Norway; Department of Genetic Technology, Ostfold County Hospital Kalnes, Sarpsborg 1714, Norway; Department of Cardiology, Ostfold County Hospital Kalnes, Sarpsborg 1714, Norway

**Keywords:** Case report, *Mycoplasma pneumoniae*, Biventricular thrombus, Cardiac arrhythmias, Cardiogenic shock, Thrombosis and cardiac imaging

## Abstract

**Background:**

This case highlights a rare and life-threatening presentation of *Mycoplasma pneumoniae* infection, demonstrating its potential to cause severe systemic complications beyond the respiratory tract. While often considered a mild illness, *M. pneumoniae* can contribute to thrombotic events, cardiac arrhythmias, and organ infarctions—complications that are under-recognized in clinical practice.

**Case summary:**

A 43-year-old man presented with cardiogenic shock caused by atrial flutter, resistant to rate-controlling medications, necessitating electrical cardioversion. Imaging revealed thrombi in both ventricles, with the left-sided thrombus posing a high embolic risk. A right-sided intracardiac mass initially suspected to be infective endocarditis was later confirmed as thrombus via computed tomography (CT) imaging, highlighting the role of advanced imaging in differential diagnosis. The patient also developed necrotizing pneumonia and splenic infarction. He was treated with antibiotics, anticoagulation, heart failure therapy, and scheduled follow-up. The aetiology of the biventricular thrombi remains unclear, likely multifactorial—either due to *M. pneumoniae*-induced hypercoagulability, biventricular dysfunction, or both.

**Conclusion:**

Physicians should be aware that *M. pneumoniae* is not always a benign infection. It can provoke severe cardiac and thrombotic complications, even in immunocompetent adults. Intracardiac thrombi, in particular, may be overlooked, and CT imaging can be an important diagnostic tool when endocarditis is suspected. Early recognition and aggressive multidisciplinary management are essential for improving outcomes in such complex presentations.

Learning points

- *Mycoplasma pneumoniae*, though often considered a mild respiratory pathogen, can cause severe systemic complications—including intracardiac thrombi and splenic infarction—that require heightened clinical awareness and prompt intervention.
- Thrombotic events associated with *M. pneumoniae* are under-recognized; physicians should maintain a high index of suspicion in atypical or severe cases to avoid delayed diagnosis and treatment.

## Introduction

*Mycoplasma pneumoniae* (*M. pneumoniae*) is a major cause of respiratory tract infections, particularly in children and young adults, accounting for 30%–40% of community-acquired pneumonia.^1^ While most infections are mild and self-limiting, *M. pneumoniae* can, in rare cases, lead to severe complications, including vasculitis, pancreatitis, myocarditis, stroke, and thrombosis.^2^

Due to the absence of a peptidoglycan cell wall, *M. pneumoniae* is inherently resistant to beta-lactam antibiotics. Macrolides, such as erythromycin or azithromycin, are the first-line treatment, while tetracyclines and fluoroquinolones serve as alternatives but have limitations in paediatric use.^3^ In Scandinavia, however, phenoxymethylpenicillin remains the standard first-line antibiotic for most acute respiratory infections,^4^ which may delay effective treatment in cases caused by *M. pneumoniae*.

**Figure 1:**
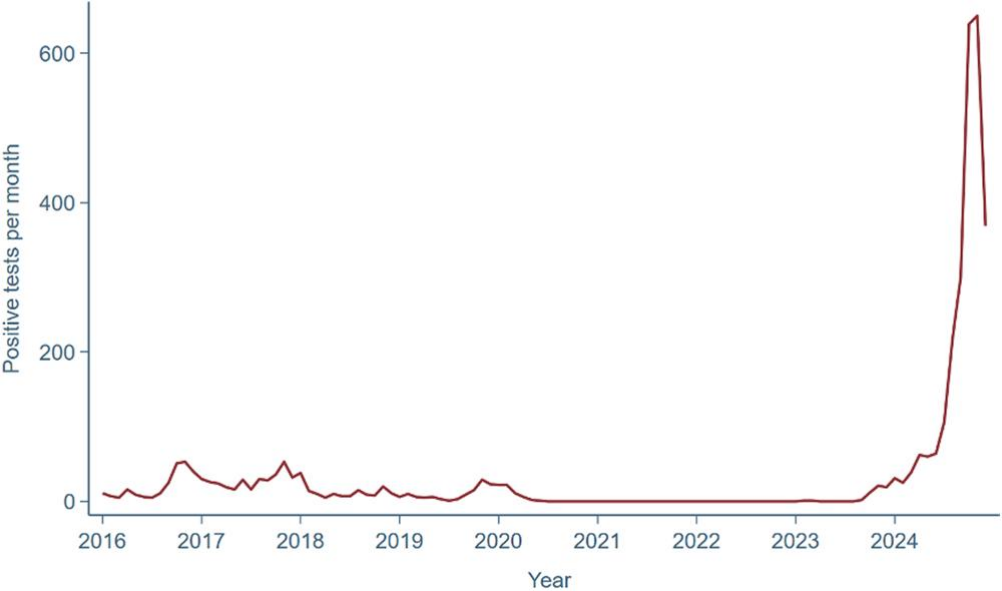
Number of positive *M. pneumoniae* polymerase chain reaction (PCR) tests per month from 2016 to 2024 (Supplementary material online, *Table S1*). Data extracted from institutional microbiology records. No individual patient data included.

Epidemics of *M. pneumoniae* typically occur in Western Europe every fourth-sixth years, driven by herd immunity dynamics, long incubation periods, and prolonged transmission rates.^5^ The last epidemic occurred in 2017–18, but restrictive measures during the COVID-19 pandemic led to an absence of cases between 2020 and 2023, followed by a sharp resurgence in late 2023 and 2024^6^ (*Figure 1*).

Here, we present the case of a 43-year-old man with *M. pneumoniae* infection complicated by necrotizing pneumonia, bilateral ventricular thrombi, and splenic infarction, an unusual and severe manifestation of this infection.

## Summary figure

**Figure:**
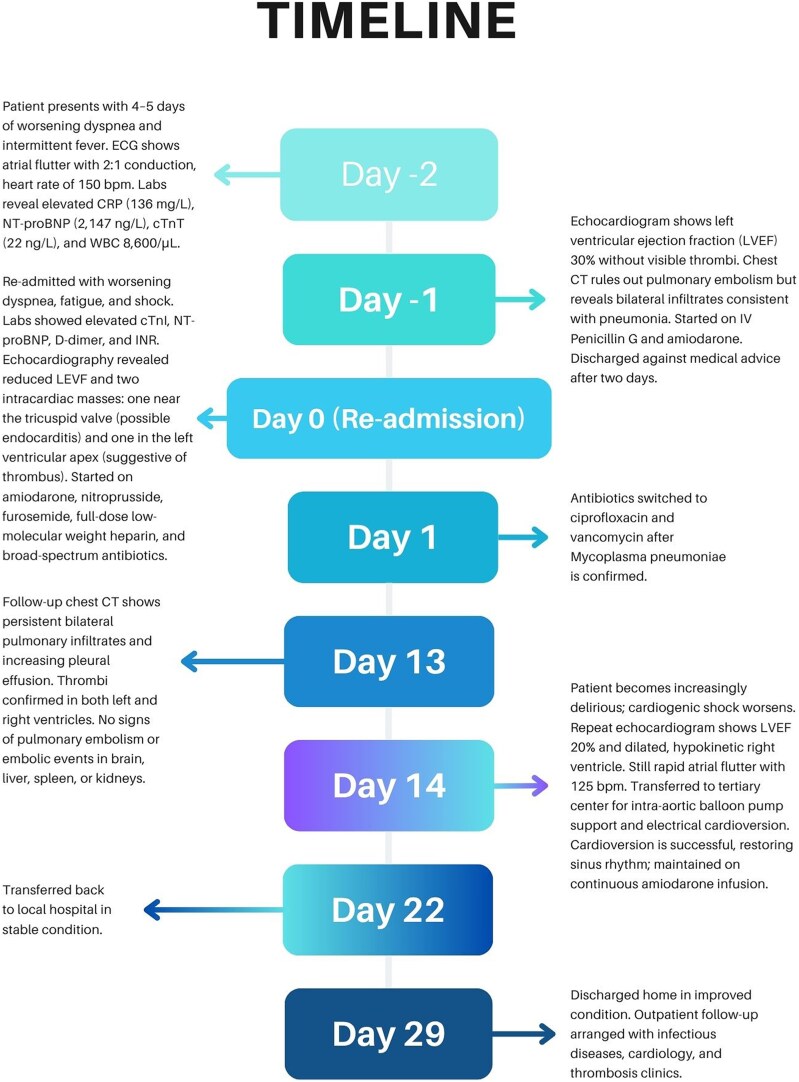


## Pathophysiology of *M. pneumoniae*

The initial effect of *M. pneumoniae* infection is local tissue and cell disruption in the bronchial epithelium. This leads to cilia loss, reduced metabolism and biosynthesis, and eventually the shedding of infected epithelial cells.^7^

Additionally, *M. pneumoniae* produces a unique virulence factor known as Community-Acquired Respiratory Distress Syndrome (CARDS) toxin, which facilitates colonization and contributes to inflammation and airway dysfunction.

Furthermore, when *M. pneumoniae* attaches to erythrocytes, hydrogen peroxide diffuses from the bacteria into the host cell, leading to glutathione depletion, lipid membrane damage, protein denaturation, heme oxidation, and haemolysis.^4,7^

One of the most serious extrapulmonary complications of *M. pneumoniae* infection is thrombosis, which can affect any part of the body and involve any vessel, leading to ischaemic events such as cerebral, splenic, or renal infarctions.^7^ Case reports have even documented aortic thrombosis and paediatric priapism.^8^ Thrombosis and its complications can develop as early as 2 days after infection or as late as 3 weeks.^3,9^

**Figure 2:**
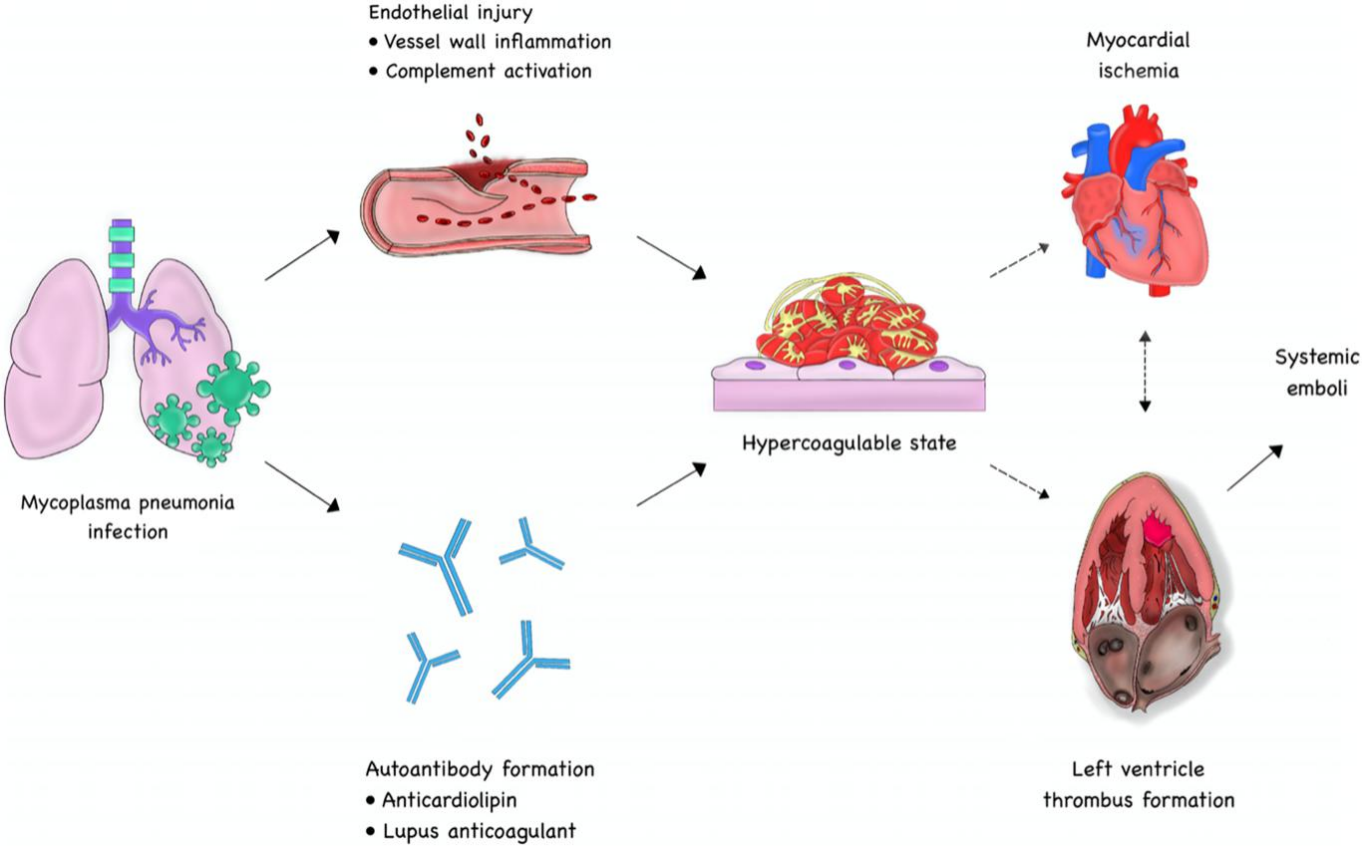
Pathophysiology of thrombus formation in *M. pneumoniae* infection. Illustration by Ai Phi Thuy Ho.

Several pathophysiological mechanisms may explain how *M. pneumoniae* causes thrombosis and vessel occlusion (*Figure 2*). First, local toxicity triggered by cytokines from lipoproteins in the bacterial cell membrane can lead to vessel damage and thrombosis without systemic hypercoagulability, primarily due to localized inflammatory reactions in the vessel walls. *M. pneumoniae* may induce the release of cytokines such as tumour necrosis factor-alpha and interleukin-8, leading to local vasculitis and thrombotic occlusion.^10^

Second, immune modulation or autoimmunity may result from cross-reactions between bacterial cell components and host cells, triggering processes such as complement activation and immune complex deposition.^2,7,10^ This mechanism can contribute to conditions like thrombotic thrombocytopenic purpura,^11^ haemolytic uraemic syndrome,^12^ or disseminated intravascular coagulation.^13^ Additionally, transient development of antiphospholipid antibodies, known to increase the risk of both venous and arterial thrombosis, has been observed.^14^

Third, seropositivity for *M. pneumoniae* and *Chlamydia pneumoniae* has been associated with an increased risk of coronary artery disease, atherosclerotic plaque instability, and myocardial infarctions.^13,14^  *Mycoplasma pneumoniae* is also frequently found in ruptured atheromas.^14,15^

These mechanisms are not mutually exclusive and may collectively contribute to thrombotic complications in *M. pneumoniae* infections. Genetic predispositions (such as thrombophilia) or environmental factors (such as drug abuse, as in our case) may further influence the severity and clinical presentation of the disease.

## Case report

A 43-year-old man with a history of drug abuse (amphetamine, cannabis, and benzodiazepines), but no known cardiovascular disease or comorbidities prior to this illness, was admitted to the nearest local hospital after 4–5 days of worsening dyspnoea and intermittent fever. An electrocardiogram revealed atrial flutter with 2:1 conduction and a ventricular rate of 150 b.p.m. Laboratory tests showed elevated inflammatory and cardiac markers, including C-reactive protein 136 mg/L, white blood cells (WBCs) at 8600 cells/µL, N-terminal pro-B-type natriuretic peptide (NT-proBNP) 2147 ng/L (reference range < 85 ng/L), and cardiac troponin T 22 ng/L (reference range 5–34 ng/L).

A chest X-ray revealed bilateral diffuse infiltrations and a small amount of pleural effusion. Initial echocardiography showed dilated left ventricle with global hypokinesia, but no regional wall abnormalities suggestive of ischaemia. The left ventricular ejection fraction (LVEF) was measured to 30% with no suspicion of intracardiac thrombi. A chest computed tomography (CT) ruled out pulmonary embolism but showed confluent bilateral pulmonary infiltrates, consistent with pneumonia.

The patient was started on penicillin G for pneumonia and received an amiodarone infusion for atrial flutter. After 1 day, penicillin G was discontinued in favour of piperacillin-tazobactam for broader coverage. However, after 2 days of intravenous antibiotics (on Day 7 after symptom onset), the patient chose to leave the hospital against medical advice. At discharge, he remained in atrial flutter with a ventricular rate of approximately 150 b.p.m., but his blood pressure remained stable, and his peripheral oxygen saturation was 94%. He was prescribed oral antibiotics (amoxicillin and ciprofloxacin). Electrical or pharmacological cardioversion was considered but declined by the patient, who also refused to undergo transoesophageal echocardiography, which was required before cardioversion given the unknown duration of arrhythmia and the risk of intracardiac thrombus. Anticoagulation was strongly recommended despite a CHA₂DS₂-VA score of 1, as his persistent atrial flutter and reduced ejection fraction placed him at increased risk of thromboembolism. The patient refused anticoagulation, expressing a strong wish to avoid all long-term medications. The medical team explained the potential risks and complications of not receiving anticoagulation, and the patient was well-informed and accepted these risks before refusing treatment.

**Figure 3:**
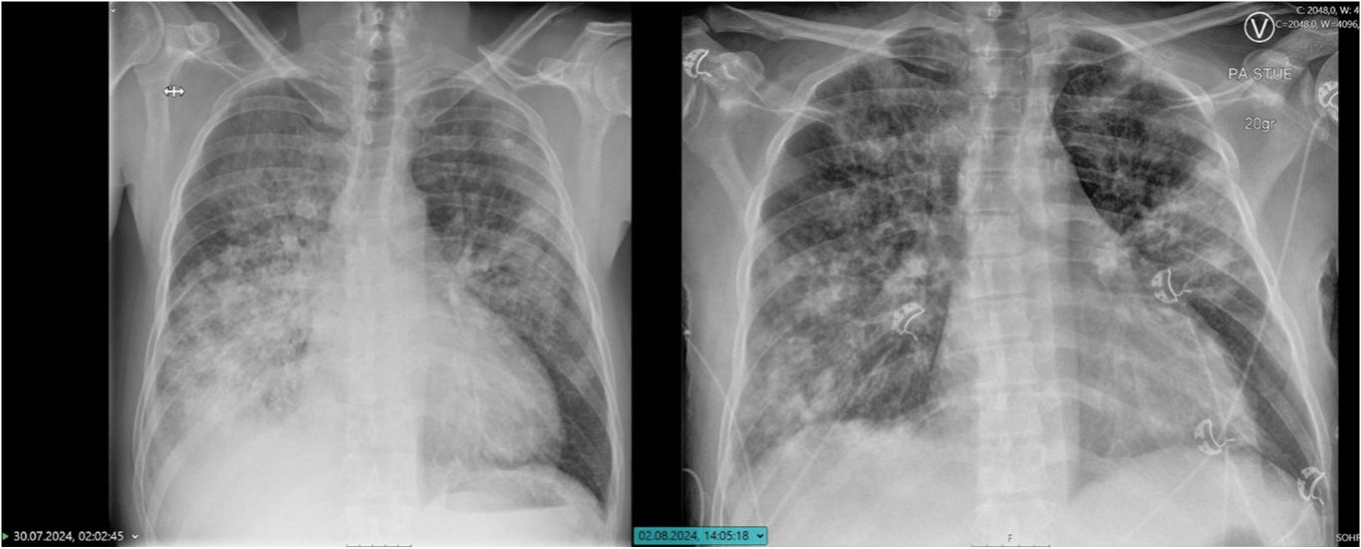
Chest X-rays from initial admission (left) and re-admission (right). The initial image shows extensive bilateral consolidations consistent with severe pneumonia and pleural effusion. On re-admission, there is persistent but slightly improved bilateral consolidation, indicating partial resolution of the pulmonary infiltrates.

Just 1 day after self-discharge (Day 8 after symptom onset), the patient was re-admitted this time to his local hospital due to progressively worsening dyspnoea and fatigue. On arrival, he was in shock, with a blood pressure of 90/55 mmHg, a respiratory rate of 28 breaths per minute, a pulse of 155 b.p.m., and a peripheral oxygen saturation of 91%. Blood gas analysis showed a PaO₂ of 8.1 kPa and a lactic acid level of 1.9 mmol/L. Chest X-ray (*Figure 3*) showed persistent bilateral consolidations, though slightly improved compared to the initial admission, indicating ongoing but partially resolving pneumonia.

Laboratory results revealed a WBC count of 22 800 cells/µL (with neutrophils at 19 600 cells/µL) and a platelet count of 134 000/µL. Cardiac markers were further elevated, with cardiac troponin I (cTnI) 40 ng/L (reference range < 34 ng/L) and NT-proBNP 3022 ng/L. The D-dimer level was >20 mg/L (reference range < 0.5), and INR was 1.3. The patient remained in atrial flutter with a ventricular rate of 150 b.p.m.

**Figure 4:**
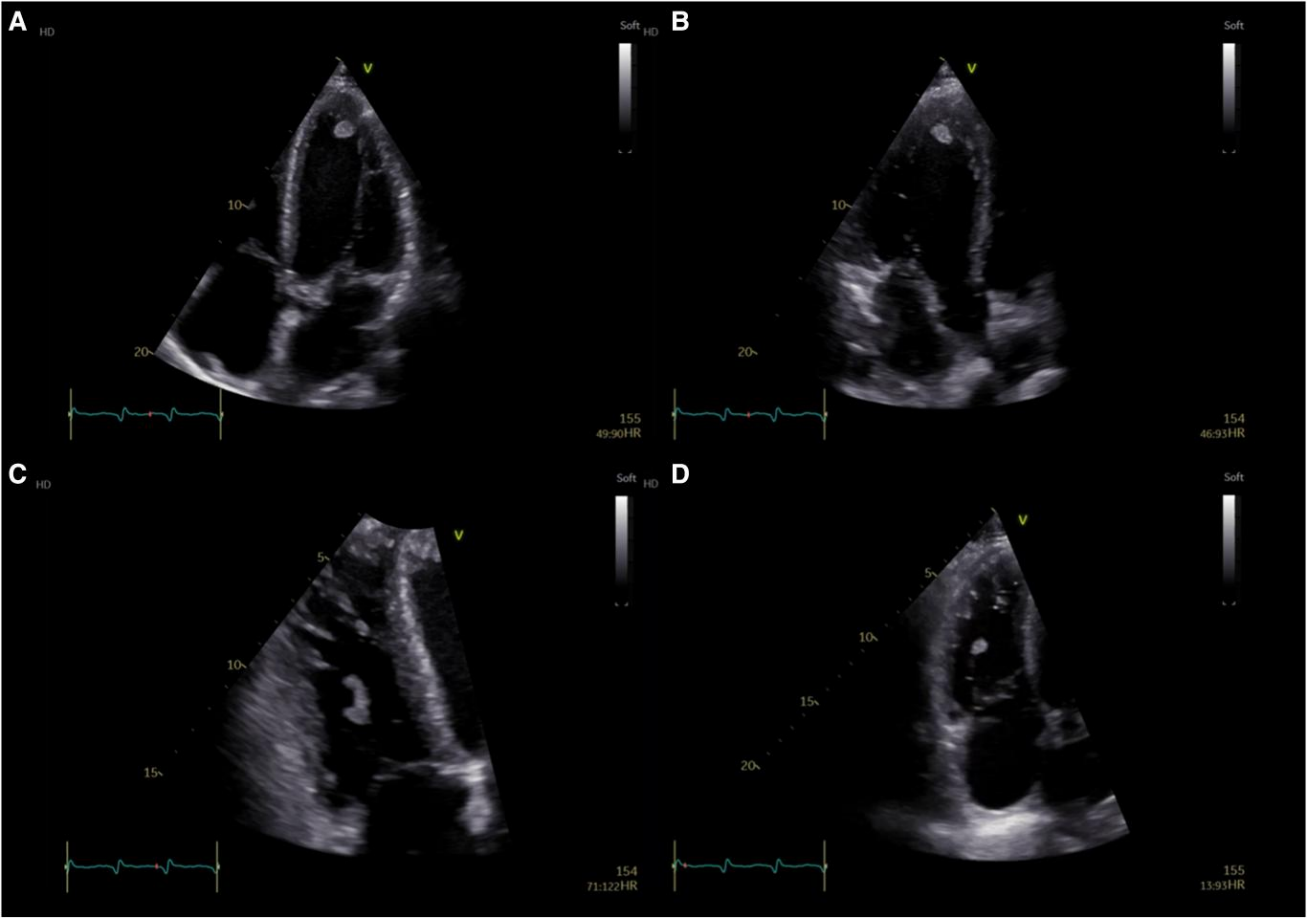
Echocardiography revealing two masses. A left ventricular mass at the apex is seen in apical four-chamber (*A*) and apical three-chamber views (*B*). The second mass is observed on the tricuspid valve chordae (*C* and *D*).

Echocardiography now showed a further decline in left ventricular function, with an LVEF of 22%. Two distinct masses were observed, one pedunculated and adherent to the tricuspid valve chordae and another located at the apex of the left ventricle (*Figure 4*). However, right ventricular systolic function remained near normal.

The patient was admitted and initiated on amiodarone, nitroprusside, and furosemide, along with a combination of cloxacillin, gentamicin, and azithromycin for antimicrobial coverage. Full-dose low molecular weight heparin (Fragmin 10 000 IU twice daily) was also started. Troponin I levels showed only a modest increase (40 ng/L on re-admission, 56 ng/L on Day 1, and 27 ng/L on Day 5), without a dynamic rise or fall, making acute myocardial infarction or myocarditis less likely.

Due to his haemodynamic instability and severely reduced left ventricular function, negatively inotropic agents such as beta-blockers or calcium channel blockers were contraindicated, as they could have led to cardiovascular collapse. A transoesophageal echocardiogram was not feasible in this setting. Consequently, intravenous amiodarone was the most appropriate option for ventricular rate control and rhythm stabilization, administered in parallel with full anticoagulation.

By the following day, the D-dimer level had decreased to 6.6 mg/L, fibrinogen was slightly elevated at 5.3 g/L (reference range 2.0–4.0 g/L), and activated partial thromboplastin time (APTT) was within the normal range at 35 s (reference range 30–42 s). On Day 13 after symptom onset (5 days after re-admission), antibiotics were switched to ciprofloxacin and vancomycin following a positive test for *M. pneumoniae*.

A follow-up chest CT on Day 13 showed persistent bilateral pulmonary infiltrates with increasing pleural effusion. Thrombi were now confirmed in both the left and right ventricles. However, there was no evidence of pulmonary embolism or embolic events affecting the brain, liver, spleen, or kidneys.

On Day 14 after the onset of symptoms (Day 6 after the second admission), the patient became increasingly delirious, and his cardiogenic shock worsened. A repeat echocardiogram revealed a LVEF of 20% and a dilated, hypokinetic right ventricle. Still in atrial flutter with a ventricular rate of 125/min, he was transferred to a tertiary care centre for intra-aortic balloon pump support and an attempt at direct current cardioversion of the atrial flutter. This intervention was performed to prevent a potentially catastrophic thromboembolic event, such as a stroke. The procedure was successful, and the patient maintained sinus rhythm with a continuous infusion of amiodarone.

He was transferred back to his local hospital on Day 22 after the onset of symptoms. Over the course of the remaining hospital stay, the patient recovered without further complications and was discharged home on Day 29. Transthoracic echocardiography showed improvement of the ejection fraction of 35%–40% before discharge.

It was concluded that the patient had suffered from necrotizing pneumonia caused by *M. pneumoniae*, leading to sepsis, atrial flutter with a rapid ventricular rate, heart failure, biventricular thrombi, and a splenic infarction. The exact cause of the biventricular thrombus formation remains uncertain, whether it was due to the prothrombotic effects of *M. pneumoniae* or reduced biventricular function, or both, is unclear. Notably, right ventricular function was not found to be impaired before Day 14, although right ventricular thrombus was observed as early as Day 8.

Unfortunately, thrombophilia testing was only performed at discharge, revealing a slightly elevated lupus anticoagulant dRVVT of 1.23 U/mL (reference range 0.88–1.21). It is likely that this value had been higher earlier in the disease course.

He was discharged with normal levels of cTnI, NT-proBNP, APTT, INR, and kidney function. His C-reactive protein was 10 mg/L, and he had mild anaemia that was normalizing at 12.9 g/dL, as well as reactive thrombocytosis with a platelet count of 759 000/µL. The medications at discharge included: edoxaban 60 mg once daily, amiodarone 200 mg once daily, ramipril 5 mg twice daily, spironolactone 12.5 mg once daily, doxycycline 100 mg twice daily, ertapenem intravenous 1 g once daily, and anidulafungin intravenous 100 mg once daily. The last three medications were to be continued for another 3 weeks.

## Discussion

Cardiac thrombus formation is a rare but serious complication of *M. pneumoniae* infection.^3,9,15^ The incidence of this complication among all individuals infected with *M. pneumoniae* is unknown. However, growing awareness of this potentially life-threatening issue has primarily arisen through case reports.^2,3,9,15–17^ Most reported cases involve younger patients. It remains unclear whether this is due to an increased likelihood of thrombotic complications resulting from immune system responses to *M. pneumoniae* in younger patients, or whether thrombotic complications are less notable in elderly patients and thus less frequently published. Additionally, it is more challenging to identify a single causative factor for thrombosis in elderly patients, whereas infection with *M. pneumoniae* is more clearly recognized as a contributing factor in younger, healthier individuals.

The complexity of immune responses to *M. pneumoniae* infections, along with the diverse and potentially life-threatening thrombotic complications, highlights the need for a more systematic approach than case reports alone can provide. Thrombotic events in *M. pneumoniae* infections, though infrequent, can be severe, suggesting an under-recognized but significant complication. Case reports, while informative, are limited in scope and do not provide a comprehensive understanding of the incidence, pathophysiology, or optimal management strategies for these complications.

Studies investigating thrombotic complications in confirmed *M. pneumoniae* infections are essential. Research should examine the incidence and types of thrombosis, including venous thromboembolism, intracardiac thrombi, and coronary thrombosis,^4^ to establish a clearer picture of how these complications manifest. Understanding whether these events result from direct prothrombotic effects of *M. pneumoniae*, immune dysregulation, or a combination is critical for developing effective prevention and treatment strategies.

Further research is essential to understand the mechanisms behind thrombus formation in *M. pneumoniae* infections. Investigating whether endothelial damage, altered coagulation profiles, or immune-mediated platelet aggregation plays a primary role is crucial for tailoring effective antithrombotic therapies. Managing right- and left-sided intracardiac thrombi requires a careful balance of anticoagulation to prevent embolic events, while in coronary thrombosis, therapy must be adjusted to mitigate both bleeding and ischaemic risks.

Large-scale studies are urgently needed to determine the incidence, risk factors, and optimal antithrombotic strategies for thrombotic complications associated with *M. pneumoniae* infections. Identifying which patient populations would benefit most from prophylactic anticoagulation is critical, but this must be weighed against potential bleeding risks.

In addition to infectious and immune mechanisms, patient-specific factors likely influenced the severity of this case. Our patient had a history of chronic substance abuse, including stimulants and benzodiazepines. While otherwise young and without known comorbidities, stimulant use and long-term drug abuse may predispose to immune dysregulation, endothelial injury, and exaggerated inflammatory responses, potentially compounding the severity of *M. pneumoniae* infection. Moreover, his poor compliance with medical treatment, including early self-discharge against medical advice, likely contributed to disease progression and delayed effective management. Together, these factors may have played a role in the development of a prothrombotic state, severe cardiac involvement, and the overall complicated clinical course observed.

Another important consideration is the use of prophylactic anticoagulation in hospitalized patients with *M. pneumoniae* infections. Key questions remain regarding the appropriate dosing and duration of therapy. Should antiphospholipid antibodies and D-dimer levels guide anticoagulation decisions, or should all patients with severe infections receive primary prophylaxis? While many young patients tolerate anticoagulation without significant bleeding risk, careful risk-benefit analysis is necessary to ensure the best outcomes.

Perhaps even more crucial is the role of early antibiotic therapy in preventing complications. Administering empiric antibiotics that cover *M. pneumoniae* could help reduce the risk of thrombosis and severe disease, particularly during seasonal outbreaks or pandemics. By treating infections early, we may be able to prevent serious complications without relying on prophylactic anticoagulation, ultimately allowing more patients to recover outside of hospital settings.

## Lead author biography


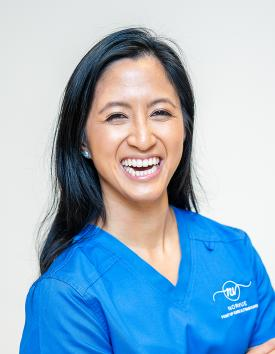


Dr Ai Phi Thuy Ho is a cardiology fellow at Ostfold County Hospital Kalnes, specializing in cardiovascular care, and has a great passion for point-of-care ultrasound (POCUS). Educated in Norway (MD, 2015), she is the founder and CEO of NorVue, a platform providing POCUS training for healthcare professionals. Dr Ai Phi Thuy Ho is passionate about advancing diagnostic accuracy and clinical education. She is also actively involved in humanitarian work across Africa and contributes to a US-based non-profit focused on global health outreach and medical teaching. Her dedication to innovation, learning, and service reflects a deep commitment to improving patient care both locally and globally.

## Supplementary Material

ytaf467_Supplementary_Data

## Data Availability

Data on number of positive tests for *M. pneumoniae* is owned by a third party and may be shared upon request. Data used in the case report are stored in electronic patient records by the third party and owned by the patient. Access to these data may be available after permission has been obtained from both the data holder and the patient.
