# Supplementary material for: Double trouble: Mycoplasma pneumoniae and its unexpected complications
Source: Eur Heart J Case Rep. 2025 Sep 18;9(10):ytaf467. doi: 10.1093/ehjcr/ytaf467 (PMC12509854; doi:10.1093/ehjcr/ytaf467)
Supplement: ytaf467_Supplementary_Data [file ytaf467_supplementary_data.docx]

**Supplementary material**

**Table S1, Number of positive tests for M. pneumoniae registered at XXXXX Hospital Trust, April 2015 through 2024**

|  | **January** | **February** | **March** | **April** | **May** | **June** | **July** | **August** | **September** | **October** | **November** | **December** |
| --- | --- | --- | --- | --- | --- | --- | --- | --- | --- | --- | --- | --- |
| **2015** |  |  |  |  |  |  |  |  |  |  |  |  |
| Number of positive tests |  |  |  | 0 | 0 | 5 | 3 | 5 | 7 | 7 | 16 | 16 |
| Total number analysed |  |  |  | 94 | 235 | 206 | 180 | 149 | 184 | 274 | 307 | 394 |
| Percentage positive |  |  |  | 0 | 0 | 2.4 | 1.7 | 3.4 | 3.8 | 2.6 | 5.2 | 4.1 |
| **2016** |  |  |  |  |  |  |  |  |  |  |  |  |
| Number of positive tests | 11 | 7 | 5 | 16 | 9 | 6 | 5 | 11 | 25 | 51 | 53 | 40 |
| Total number analysed | 471 | 650 | 537 | 370 | 390 | 296 | 214 | 354 | 435 | 534 | 779 | 1119 |
| Percentage positive | 2.3 | 1.1 | 0.9 | 4.3 | 2.3 | 2 | 2.3 | 3.1 | 5.7 | 9.6 | 6.8 | 3.6 |
| **2017** |  |  |  |  |  |  |  |  |  |  |  |  |
| Number of positive tests | 30 | 26 | 24 | 19 | 16 | 29 | 16 | 30 | 28 | 36 | 53 | 32 |
| Total number analysed | 1113 | 797 | 735 | 479 | 600 | 547 | 377 | 435 | 499 | 637 | 747 | 1052 |
| Percentage positive | 2.7 | 3.3 | 3.3 | 4 | 2.7 | 5.3 | 4.2 | 6.9 | 5.6 | 5.7 | 7.1 | 3 |
| **2018** |  |  |  |  |  |  |  |  |  |  |  |  |
| Number of positive tests | 38 | 14 | 10 | 5 | 10 | 7 | 7 | 15 | 9 | 8 | 20 | 11 |
| Total number analysed | 1365 | 1236 | 1086 | 784 | 682 | 602 | 426 | 551 | 710 | 833 | 863 | 958 |
| Percentage positive | 2.8 | 1.1 | 0.9 | 0.6 | 1.5 | 1.2 | 1.6 | 2.7 | 1.3 | 1 | 2.3 | 1.1 |
| **2019** |  |  |  |  |  |  |  |  |  |  |  |  |
| Number of positive tests | 6 | 10 | 6 | 5 | 6 | 3 | 1 | 3 | 9 | 15 | 29 | 23 |
| Total number analysed | 1513 | 1506 | 1022 | 816 | 763 | 703 | 555 | 548 | 680 | 876 | 918 | 1023 |
| Percentage positive | 0.4 | 0.7 | 0.6 | 0.6 | 0.8 | 0.4 | 0.2 | 0.5 | 1.3 | 1.7 | 3.2 | 2.2 |
| **2020** |  |  |  |  |  |  |  |  |  |  |  |  |
| Number of positive tests | 22 | 22 | 11 | 6 | 2 | 1 | 0 | 0 | 0 | 0 | 0 | 0 |
| Total number analysed | 1324 | 1167 | 1113 | 633 | 236 | 187 | 224 | 238 | 261 | 252 | 296 | 261 |
| Percentage positive | 1.7 | 1.9 | 1 | 0.9 | 0.8 | 0.5 | 0 | 0 | 0 | 0 | 0 | 0 |
| **2021** |  |  |  |  |  |  |  |  |  |  |  |  |
| Number of positive tests | 0 | 0 | 0 | 0 | 0 | 0 | 0 | 0 | 0 | 0 | 0 | 0 |
| Total number analysed | 224 | 167 | 210 | 150 | 148 | 144 | 169 | 174 | 355 | 685 | 842 | 481 |
| Percentage positive | 0 | 0 | 0 | 0 | 0 | 0 | 0 | 0 | 0 | 0 | 0 | 0 |
| **2022** |  |  |  |  |  |  |  |  |  |  |  |  |
| Number of positive tests | 0 | 0 | 0 | 0 | 0 | 0 | 0 | 0 | 0 | 0 | 0 | 0 |
| Total number analysed | 325 | 297 | 481 | 475 | 441 | 425 | 259 | 327 | 415 | 442 | 608 | 773 |
| Percentage positive | 0 | 0 | 0 | 0 | 0 | 0 | 0 | 0 | 0 | 0 | 0 | 0 |
| **2023** |  |  |  |  |  |  |  |  |  |  |  |  |
| Number of positive tests | 0 | 1 | 1 | 0 | 0 | 0 | 0 | 0 | 2 | 12 | 21 | 19 |
| Total number analysed | 869 | 582 | 573 | 314 | 355 | 298 | 207 | 287 | 431 | 550 | 848 | 773 |
| Percentage positive | 0 | 1.2 | 1.2 | 0 | 0 | 0 | 0 | 0 | 0.5 | 2.2 | 2.5 | 2.5 |
| **2024** |  |  |  |  |  |  |  |  |  |  |  |  |
| Number of positive tests | 31 | 25 | 39 | 62 | 60 | 64 | 106 | 217 | 301 | 639 | 650 | 369 |
| Total number analysed | 1040 | 974 | 763 | 742 | 737 | 867 | 795 | 1333 | 1893 | 3020 | 3576 | 2756 |
| Percentage positive | 3 | 2.6 | 5.1 | 8.4 | 8.1 | 7.4 | 13.3 | 16.3 | 15.9 | 21.2 | 18.2 | 13.4 |
